# Upper airway resistance syndrome in polycystic ovary syndrome: a narrative literature review

**DOI:** 10.1590/1806-9282.20260437

**Published:** 2026-08-24

**Authors:** Tugba Karagoz, Warren Bennett, Atakan Tanacan

**Affiliations:** 1 University Hospitals Bristol and Weston NHS Foundation Trust, Department of Otorhinolaryngology – Bristol, United Kingdom.; 2 Ministry of Health, Ankara City Hospital, Department of Obstetrics and Gynecology – Ankara, Turkey.

## INTRODUCTION

Polycystic ovary syndrome (PCOS) is the most prevalent endocrine disorder in reproductive-aged women, affecting 8–13% of this population worldwide^1^. Characterized by hyperandrogenism, ovulatory dysfunction, and polycystic ovarian morphology, PCOS extends beyond reproductive manifestations to encompass significant metabolic and cardiovascular comorbidities^2^. The association between PCOS and obstructive sleep apnea (OSA) is well-established, with prevalence rates substantially higher than in age- and weight-matched controls^3^. Longitudinal data from Brazilian cohorts further confirm that PCOS phenotype influences the trajectory of metabolic syndrome, underscoring the importance of recognizing all metabolic comorbidities, including sleep-disordered breathing (SDB), in this population.

However, upper airway resistance syndrome (UARS)—a distinct SDB phenotype characterized by increased upper airway resistance, sleep fragmentation, and excessive daytime sleepiness without significant oxygen desaturation or frank apneas—remains critically underrecognized in PCOS patients^4^. First described by Guilleminault et al. in 1993, UARS occupies a unique position in the SDB spectrum, bridging primary snoring and OSA^5^. Unlike OSA, which predominantly affects older, obese males with witnessed apneas and loud snoring, UARS typically presents in younger, non-obese individuals, particularly women, with atypical symptoms such as insomnia, fatigue, and morning headaches^6^.

The pathophysiological overlap between PCOS and UARS is substantial. Hyperandrogenism may promote upper airway soft tissue deposition and pharyngeal collapsibility^7^. Progesterone deficiency, common in anovulatory PCOS, is hypothesized to reduce ventilatory drive and upper airway dilator muscle tone^8^. Insulin resistance and chronic low-grade inflammation—hallmarks of PCOS—may contribute to sympathetic nervous system overactivity and sleep fragmentation^9^. Despite these mechanistic links, UARS in PCOS remains underdiagnosed due to its atypical presentation and the requirement for specialized diagnostic techniques, particularly esophageal pressure (Pes) monitoring^10^. The potential role of environmental endocrine disruptors such as bisphenol A (BPA) in PCOS pathogenesis may further modulate metabolic and hormonal pathways relevant to SDB, though this remains speculative.

This narrative literature review synthesizes current evidence on UARS in PCOS, emphasizing its unique pathophysiology, clinical presentation, diagnostic requirements, and treatment strategies. Recognition of UARS as a distinct phenotype in PCOS is essential for comprehensive patient care and prevention of long-term cardiometabolic sequelae.

## METHODS

This is a narrative literature review designed to synthesize current evidence on UARS in PCOS. This review did not follow PRISMA guidelines, as it is a narrative review, not a systematic review. A comprehensive literature search was conducted across PubMed and Google Scholar, covering publications from 2000 to 2024. Search terms included combinations of "polycystic ovary syndrome," "PCOS," "upper airway resistance syndrome," "UARS," "sleep-disordered breathing," "respiratory effort-related arousals," "RERAs," "esophageal manometry," "hyperandrogenism," and "insulin resistance."

Inclusion criteria encompassed: (1) original research articles reporting SDB or UARS prevalence in PCOS populations; (2) mechanistic studies investigating hormonal, metabolic, or anatomical factors linking PCOS to upper airway dysfunction; (3) diagnostic studies utilizing polysomnography (PSG) with or without esophageal pressure monitoring; (4) treatment studies evaluating interventions for SDB in PCOS; and (5) systematic reviews and meta-analyses addressing PCOS–SDB associations.

Exclusion criteria: Studies were excluded if they: (1) lacked clear diagnostic criteria for PCOS or SDB; (2) focused exclusively on pediatric or postmenopausal populations; (3) were case reports with fewer than five subjects; or (4) were not available in English.

Data extraction focused on study design, sample characteristics, SDB prevalence and phenotype, diagnostic methods, pathophysiological mechanisms, treatment interventions, and clinical outcomes. Particular attention was paid to studies distinguishing UARS from OSA and those employing esophageal pressure monitoring. Given the narrative nature of this review, formal quality assessment and meta-analysis were not performed. Evidence synthesis prioritized recent publications (2021–2024) while incorporating foundational studies establishing key concepts.

## RESULTS

### Prevalence and clinical burden

Importantly, no published study was identified that directly reported UARS prevalence in PCOS using esophageal pressure (Pes) monitoring. The evidence base, therefore, relies on extrapolation from OSA studies in PCOS, mechanistic investigations, and UARS studies in general populations.

Sleep-disordered breathing is substantially more prevalent in women with PCOS compared to matched controls. Vgontzas et al.^3^ demonstrated that 17% of PCOS patients exhibited SDB [defined as apnea-hypopnea index (AHI) ≥5] compared to 4% of controls, with daytime sleepiness representing a stronger predictor than body mass index (BMI). Subsequent studies using PSG have reported SDB prevalence ranging from 40–56% in PCOS cohorts^9,11,12^. The wide range in reported SDB prevalence (17–56%) reflects methodological heterogeneity across studies, including differing PCOS diagnostic criteria (Rotterdam vs. NIH), variable BMI distributions in study cohorts, and inconsistent polysomnographic definitions of SDB (AHI ≥5 vs. ≥15 events/hour). Studies enrolling predominantly obese PCOS cohorts report higher SDB prevalence, while community-based cohorts with leaner participants report lower rates. Population-based cohort studies confirm this association, with Kumarendran et al.^13^ reporting hazard ratios of 1.5–2.0 for OSA diagnosis in PCOS patients over time. This finding is corroborated by a meta-analysis by Helvaci et al., who reported significantly increased odds of obstructive sleep apnea among women with PCOS compared with controls, further reinforcing the consistency of the PCOS–OSA association across independent samples and study designs^14^.

However, these prevalence estimates predominantly capture moderate-to-severe OSA with significant oxygen desaturation. UARS, characterized by respiratory effort-related arousals without substantial desaturation, requires esophageal pressure monitoring for accurate diagnosis and is likely underrepresented in standard PSG studies^15^.

Table 1 summarizes key studies informing the evidence base for PCOS–UARS associations, including study design, sample characteristics, and principal findings relevant to sleep-disordered breathing in PCOS.

**Table 1:** Summary of key studies informing the polycystic ovary syndrome–upper airway resistance syndrome evidence base.

Author	Design	n	PCOS criteria	SDB definition	Key finding relevant to PCOS-UARS
Vgontzas et al.^3^	Cross-sectional	53 PCOS, 452 controls	NIH	AHI≥5	SDB in 17% PCOS vs. 4% controls; daytime sleepiness stronger predictor than BMI
Fogel et al.^11^	Cross-sectional	44 obese PCOS	NIH	AHI≥5	Elevated OSA prevalence in obese PCOS; androgens correlate with OSA severity. Note: obese cohort with established OSA; not UARS-specific
Tasali et al.^12^	Cross-sectional	40 PCOS	Rotterdam	AHI≥5	OSA in 70% of obese PCOS; insulin resistance mediates association
Kumarendran et al.^13^	Longitudinal cohort	2,543 PCOS	UK primary care codes	OSA diagnosis	HR 1.5–2.0 for incident OSA in PCOS vs. controls
Helvaci et al. ^14^	Meta-analysis	Multiple studies	Various	OSA diagnosis	Significantly increased odds of OSA in women with PCOS compared with controls, supporting the prevalence estimates above
Yang et al.^9^	Cross-sectional	302 PCOS	Rotterdam	AHI ≥5	SDB prevalence 40–56%; hyperandrogenism independently associated
Guilleminault et al.^10^	Case series	40	N/A (general population)	Pes-confirmed RERAs	UARS presents with chronic fatigue and unrefreshing sleep; Pes gold standard for diagnosis. Extrapolated to PCOS context
Popovic and White^8^	Experimental	12 normal women	N/A	Upper airway muscle EMG	Progesterone increases upper airway dilator muscle activity. Normal women across menstrual cycle; not PCOS patients
Tasali et al.^21^	RCT	21 obese PCOS	Rotterdam	AHI ≥5	CPAP improves insulin sensitivity in OSA-PCOS. OSA study; not UARS-specific
Lim et al.^20^	Cochrane review	Multiple RCTs	Various	AHI/SDB	Lifestyle modification improves SDB and metabolic parameters in PCOS
Camacho et al.^23^	Systematic review	9 studies	N/A	Snoring/SDB	Oropharyngeal exercises significantly reduce snoring and mild SDB; emerging evidence for UARS

PCOS: polycystic ovary syndrome; UARS: upper airway resistance syndrome; OSA: obstructive sleep apnea; SDB: sleep-disordered breathing; AHI: apnea-hypopnea index; Pes: esophageal pressure; HR: hazard ratio; RCT: randomized controlled trial; N/A: not applicable; BMI: body mass index; RERAs: respiratory effort-related arousals; CPAP: continuous positive airway pressure; NIH: National Institutes of Health; EMG: electromyography.

### Pathophysiological mechanisms

The pathophysiology linking PCOS to UARS involves multiple interconnected mechanisms.

Hyperandrogenism and Upper Airway Anatomy: Elevated androgens may promote fat deposition in pharyngeal tissues, increasing upper airway collapsibility. In obese PCOS patients with established OSA, testosterone levels have been shown to correlate with OSA severity independent of BMI^11^; however, it should be noted that this finding derives from a study of obese PCOS patients with frank OSA, not UARS specifically. Whether androgen-mediated anatomical changes contribute to the subtler increases in airway resistance characteristic of UARS—as opposed to frank apneas—remains to be directly investigated. Androgens are also hypothesized to influence craniofacial morphology, with PCOS patients demonstrating narrower upper airways and increased pharyngeal soft tissue volume on imaging studies^16^.

Progesterone Deficiency: Progesterone acts as a respiratory stimulant and enhances upper airway dilator muscle activity. Anovulatory PCOS patients with chronic progesterone deficiency may exhibit reduced ventilatory drive and increased susceptibility to upper airway collapse during sleep. This mechanism is particularly relevant to UARS, where subtle increases in airway resistance trigger arousal responses. It is important to emphasize that the cited study by Popovic and White^8^ examined normal women across the menstrual cycle, not PCOS patients. Extrapolation of these findings to PCOS-UARS, while mechanistically plausible, represents an unvalidated inference that requires direct empirical investigation in PCOS cohorts.

Insulin Resistance and Metabolic Dysfunction: Insulin resistance, present in 50–70% of PCOS patients regardless of weight, may contribute to SDB through multiple pathways. Hyperinsulinemia is hypothesized to promote sympathetic nervous system activation, increasing sleep fragmentation and arousal frequency. Chronic inflammation associated with insulin resistance may lead to upper airway edema and increased resistance. The claim regarding leptin resistance impairing central respiratory drive, while biologically plausible, lacks direct citation support specific to PCOS-UARS interactions^17^. Additionally, the study by Vgontzas et al.^7^ demonstrating elevated interleukin-6 (IL-6) in PCOS addressed inflammation in PCOS patients without sleep apnea, not upper airway anatomical changes per se; this extrapolation should be interpreted accordingly.

Autonomic Dysfunction: PCOS patients demonstrate increased sympathetic tone and reduced heart rate variability, even in the absence of diagnosed SDB^18^. Sleep fragmentation from RERAs further amplifies sympathetic activation, creating a vicious cycle of metabolic deterioration and worsening upper airway function.

### Clinical presentation and diagnostic challenges

UARS in PCOS presents distinct clinical features that differ from classical OSA.

Atypical Symptomatology: Rather than loud snoring and witnessed apneas, UARS patients report insomnia, difficulty maintaining sleep, unrefreshing sleep, morning headaches, and profound daytime fatigue^6^. These symptoms overlap substantially with common PCOS complaints, leading to frequent misattribution and delayed diagnosis.

Demographic Profile: UARS typically affects younger, non-obese women—precisely the demographic overrepresented in PCOS populations^6^. This contrasts with the older, obese, male-predominant OSA phenotype, contributing to clinical underrecognition.

Importantly, fatigue, insomnia, and morning headaches are not specific to UARS. These symptoms overlap extensively with other conditions highly prevalent in PCOS populations, including chronic fatigue syndrome, depression, hypothyroidism and other thyroid dysfunction, and iron deficiency anemia. Clinicians should systematically exclude these common confounders through thyroid function testing, complete blood count, ferritin measurement, and mental health screening before attributing symptoms exclusively to UARS. Failure to do so may result in missed diagnoses of treatable comorbidities and inappropriate attribution of symptoms to sleep-disordered breathing.

The following clinical pearl box provides a practical framework for differential diagnosis in PCOS patients presenting with fatigue, insomnia, or morning headaches before referral for sleep evaluation.

Clinical recommendation: In PCOS patients presenting with fatigue, insomnia, or morning headaches, a stepwise approach is recommended: (1) screen and treat depression, thyroid dysfunction, and iron deficiency first; (2) if symptoms persist despite adequate treatment, proceed to PSG; and (3) if standard PSG shows AHI <5 with persistent symptoms, consider Pes-based monitoring or detailed nasal pressure flow curve analysis for RERA detection.

Diagnostic requirements: Standard PSG may appear normal or show only mild abnormalities in UARS patients. The AHI is often <5 events/hour, and oxygen desaturation is minimal^15^. Diagnosis requires identification of RERAs—events characterized by increasing respiratory effort terminated by arousal without meeting criteria for apnea or hypopnea.

While esophageal pressure (Pes) monitoring remains the research gold standard for RERA detection, Pes is invasive, poorly tolerated, and rarely used in routine clinical practice. Current American Academy of Sleep Medicine (AASM) guidelines permit nasal pressure transducer signals as a surrogate for RERA identification^19^. However, nasal pressure transducers have substantially lower sensitivity than Pes for detecting RERAs, and many sleep laboratories do not routinely score RERAs even when nasal pressure signals are available. This contributes to the systematic underdiagnosis of UARS in routine clinical practice. Cyclical changes in the nasal pressure flow curve contour—such as flattening or flow limitation—may also suggest increased airway resistance and can be used to identify RERAs in the absence of Pes monitoring^10,19^.

Respiratory disturbance index (RDI): The RDI, which includes apneas, hypopneas, and RERAs, provides a more accurate assessment of SDB burden in UARS. An RDI ≥5 with AHI <5 suggests UARS^15^. However, esophageal manometry is not routinely performed in most sleep laboratories, leading to systematic underdiagnosis.

### Treatment approaches

Important caveat: No randomized controlled trials (RCTs) have evaluated any intervention specifically for UARS in PCOS. The evidence presented below is therefore graded by source strength as follows: (Level 1) evidence from RCTs or Cochrane reviews in PCOS-OSA; (Level 2) evidence extrapolated from OSA studies in PCOS; (Level 3) evidence extrapolated from general UARS populations; (Level 4) theoretical/mechanism-based reasoning. Readers should interpret all treatment recommendations in this context.

Management of UARS in PCOS requires strategies addressing both conditions.

Lifestyle Modification (Level 1—RCT/Cochrane evidence in PCOS-SDB): Weight loss through diet and exercise provides dual benefits, improving both metabolic parameters and upper airway function^20^. Even modest weight reduction (5–10% of body weight) significantly reduces SDB severity and improves insulin sensitivity in PCOS patients, as demonstrated in a Cochrane systematic review^20^. However, weight loss is challenging in PCOS due to underlying metabolic dysfunction, and adherence rates are suboptimal. This represents the strongest available evidence base for SDB management in PCOS.

Continuous positive airway pressure (CPAP) (Level 2—extrapolated from OSA in PCOS): CPAP effectively eliminates RERAs and restores sleep architecture in UARS patients. The cited evidence for CPAP improving insulin sensitivity and cardiovascular function in PCOS derives from an RCT in OSA, not UARS specifically^21^. No RCTs of CPAP for UARS in PCOS exist. While mechanistic extrapolation suggests benefit, the magnitude of metabolic improvement in UARS—where hypoxemia is minimal—may differ substantially from that observed in OSA. CPAP does not improve reproductive outcomes or hyperandrogenism^22^. Adherence is particularly challenging in UARS patients due to lower perceived symptom severity and younger age.

Mandibular advancement devices (MADs) (Level 3—extrapolated from general UARS/mild SDB populations): MADs represent a well-tolerated alternative to CPAP, particularly in younger women who may find CPAP adherence challenging. MADs work by advancing the mandible and tongue, thereby increasing upper airway caliber and reducing collapsibility. RCT evidence specific to UARS in PCOS is lacking; MADs have demonstrated efficacy in mild-to-moderate SDB in general populations and may be appropriate first-line alternatives for UARS patients who are non-obese, younger, and have favorable craniofacial anatomy. Dental evaluation and custom fitting are essential for optimal outcomes.

Myofunctional Therapy (Level 3—extrapolated from general SDB populations): Oropharyngeal exercises (myofunctional therapy) targeting tongue posture, swallowing patterns, and pharyngeal muscle tone represent an emerging adjunctive treatment for mild SDB. A systematic review and meta-analysis by Camacho et al. demonstrated that oropharyngeal exercises significantly reduce snoring frequency and intensity and may reduce AHI in mild-to-moderate SDB in adults^23^. While evidence in UARS-specific populations is limited, myofunctional therapy may be considered as part of a multimodal approach, particularly in patients seeking non-device alternatives or as adjuncts to MAD or CPAP therapy.

PCOS-Specific Therapies (Level 4—theoretical/mechanism-based): Metformin improves insulin sensitivity but does not consistently improve SDB parameters. The cited reference (Shatwan et al., 2014^24^) addresses metformin and sleep quality in adolescent PCOS but does not specifically address UARS; this extrapolation should be interpreted with caution. Anti-androgen therapies (spironolactone, cyproterone acetate) address hyperandrogenism but have not demonstrated significant effects on upper airway function^25^. Oral contraceptives regulate menstrual cycles and may improve progesterone-mediated respiratory drive, but lack robust evidence for SDB improvement.

Surgical Interventions (Level 2—extrapolated from OSA-PCOS data): Bariatric surgery in obese PCOS patients with SDB produces substantial improvements in both metabolic parameters and SDB severity^26^. Upper airway surgery (uvulopalatopharyngoplasty, maxillomandibular advancement) may benefit selected patients with anatomical abnormalities, though evidence specific to PCOS-UARS is limited.

Integrated Management (Level 4—expert consensus): Current evidence supports simultaneous, integrated treatment of both PCOS and UARS rather than sequential approaches^27^. Multidisciplinary care involving endocrinologists, gynecologists, otolaryngologists, and sleep medicine specialists optimizes outcomes and addresses the complex interplay between metabolic, hormonal, and respiratory dysfunction.

## DISCUSSION

This review highlights UARS as a critically underrecognized SDB phenotype in women with PCOS. The convergence of hyperandrogenism, progesterone deficiency, and insulin resistance is hypothesized to create a unique pathophysiological milieu predisposing to increased upper airway resistance and sleep fragmentation. Unlike classical OSA, UARS presents with atypical symptoms in younger, often non-obese women, leading to frequent misdiagnosis or delayed recognition.

The diagnostic challenge posed by UARS cannot be overstated. Standard PSG without esophageal pressure monitoring systematically underdiagnoses UARS, as RERAs are difficult to identify using nasal pressure or thermistor signals alone^10^. The limited availability of esophageal manometry in routine clinical practice perpetuates this diagnostic gap.

Clinicians should suspect UARS in PCOS patients presenting with persistent fatigue, unrefreshing sleep, or morning headaches despite a normal AHI (<5) and absence of significant oxygen desaturation on standard PSG. In such cases, full-night PSG with Pes monitoring—or, where unavailable, careful review of nasal pressure flow curve morphology (looking for flow limitation and flattening)—is recommended before attributing symptoms to other PCOS comorbidities. As outlined in Table 2, systematic exclusion of thyroid dysfunction, iron deficiency anemia, depression, and chronic fatigue syndrome is essential before definitively attributing symptoms to UARS.

**Table 2:** Clinical pearl: differential diagnosis of fatigue, insomnia, and morning headaches in polycystic ovary syndrome before pursuing upper airway resistance syndrome evaluation.

Condition	Key overlapping symptoms	Distinguishing features	Recommended tests	Red flags suggesting UARS
Depression/anxiety	Fatigue, insomnia, poor concentration	Low mood, anhedonia, and anxiety symptoms predominate	PHQ-9, GAD-7, mental health screening	Symptoms persist despite adequate mental health treatment
Hypothyroidism	Fatigue, weight gain, cold intolerance, cognitive slowing	Bradycardia, dry skin, constipation	TSH, free T4	Normal thyroid function with persistent unrefreshing sleep
Iron deficiency anemia	Fatigue, headaches, reduced exercise tolerance	Pallor, pica, restless legs	FBC, serum ferritin, transferrin saturation	Normal hematology with persistent sleep fragmentation
Chronic fatigue syndrome	Profound fatigue, post-exertional malaise, unrefreshing sleep	Post-exertional worsening, cognitive dysfunction	Clinical diagnosis (NICE/CDC criteria)	AHI <5 on PSG with confirmed RERAs on Pes monitoring
UARS	Fatigue, insomnia, morning headaches, unrefreshing sleep	Normal AHI (<5), no significant desaturation, flow limitation on nasal pressure curve	Full-night PSG with nasal pressure; Pes monitoring if available	RDI ≥5 with AHI <5; flow limitation pattern; arousals without apneas/hypopneas

PHQ-9: Patient Health Questionnaire-9; GAD-7: Generalized Anxiety Disorder-7; TSH: thyroid-stimulating hormone; FBC: full blood count; AHI: apnea-hypopnea index; PSG: polysomnography; Pes: esophageal pressure; RDI: respiratory disturbance index; RERA: respiratory effort-related arousal; UARS: upper airway resistance syndrome; NICE: National Institute for Health and Care Excellence; CDC: Centers for Disease Control and Prevention.

The long-term implications of untreated UARS in PCOS are substantial. Chronic sleep fragmentation amplifies insulin resistance, promotes cardiovascular dysfunction, and exacerbates metabolic syndrome features^9^. This is consistent with the broader recognition of sleep apnea as a manifestation of the metabolic syndrome, in which sleep fragmentation and intermittent hypoxemia act as independent contributors to metabolic dysregulation^28^. Early recognition and treatment of UARS may interrupt this pathophysiological cascade and reduce long-term cardiometabolic risk.

Treatment of UARS in PCOS requires nuanced, integrated approaches. While CPAP effectively addresses upper airway resistance, it does not improve reproductive or hyperandrogenic features of PCOS^22^. Conversely, PCOS-specific therapies improve metabolic and reproductive parameters but have a limited impact on SDB^24,25^. This bidirectional independence underscores the necessity for simultaneous, multidisciplinary management rather than sequential treatment of individual conditions.

A recent global consensus process has proposed renaming polycystic ovary syndrome as "polyendocrine metabolic ovarian syndrome" (PMOS) to better reflect its multisystem endocrine and metabolic nature^29^. While this nomenclature remains under debate and has not yet been formally adopted by major guidelines, it is noteworthy in that it explicitly acknowledges PCOS/PMOS as a systemic metabolic condition extending beyond reproductive dysfunction. Within this framework, UARS can be understood as one of several extra-reproductive manifestations, reinforcing the need for multidisciplinary assessment that includes sleep evaluation. The clinical and research implications of this potential reclassification for sleep medicine remain to be determined.

In the absence of direct prevalence data, this review should be interpreted as a hypothesis-generating synthesis rather than an evidence-based prevalence estimate. The mechanistic plausibility of UARS in PCOS is strong, given the hormonal, metabolic, and anatomical factors reviewed above. However, the true burden of UARS in PCOS remains unknown. Future prospective studies using Pes-based PSG in well-characterized PCOS cohorts are needed to establish prevalence, identify risk factors, and evaluate treatment efficacy.

Three recent studies provide important contextual support for the clinical relevance of this review. Soares-Jr et al. conducted a six-year longitudinal follow-up of Brazilian women with PCOS and demonstrated that metabolic syndrome trajectory differs significantly across PCOS phenotypes, with hyperandrogenic phenotypes carrying the highest cardiometabolic burden^30^. This underscores the importance of phenotype-stratified risk assessment in PCOS—including SDB evaluation—as metabolic comorbidities accumulate differentially over time. Urbanetz et al. explored the potential role of BPA, an environmental endocrine disruptor, in PCOS pathogenesis, raising the possibility that exogenous hormonal disruptors may contribute to the hyperandrogenic and metabolic milieu that predisposes to upper airway dysfunction^31^. Finally, Camacho et al. demonstrated in a systematic review and meta-analysis that oropharyngeal exercises (myofunctional therapy) significantly reduce snoring frequency and intensity and may reduce the apnea-hypopnea index in mild-to-moderate SDB^23^. Taken together, these findings reinforce the multidimensional nature of PCOS-related sleep dysfunction and support the integration of phenotype-aware, environmentally informed, and non-pharmacological treatment strategies into the clinical management of UARS in PCOS.

## LIMITATIONS

This narrative review has several important limitations that must be acknowledged:

No published study has directly measured UARS prevalence in PCOS using esophageal pressure (Pes) monitoring. All prevalence estimates are extrapolated from OSA studies or inferred from mechanistic plausibility. The true prevalence of UARS in PCOS, therefore, remains unknown.

No UARS-specific RCTs exist in PCOS populations. Treatment recommendations are based on OSA studies in PCOS, UARS studies in general populations, or mechanistic reasoning. The efficacy of CPAP, MAD, or other interventions specifically for UARS in PCOS has not been directly evaluated.

Most mechanistic evidence derives from OSA studies in PCOS, not UARS. While the pathophysiological overlap is substantial, UARS differs from OSA in the absence of significant hypoxemia and apneas. The extent to which OSA-derived mechanisms apply to UARS remains uncertain.

Small sample sizes and heterogeneous PCOS diagnostic criteria across included studies limit generalizability. PCOS phenotypes vary widely (hyperandrogenic vs. metabolic vs. ovulatory), and SDB risk may differ across phenotypes. Most studies did not stratify by PCOS phenotype.

Possible publication bias favoring positive findings. Studies reporting associations between PCOS and SDB are more likely to be published than null findings, potentially inflating perceived effect sizes.

Inability to exclude confounding by BMI, age, and comorbidities in reviewed studies. Many studies did not adequately control for obesity, which is both a feature of many PCOS cases and an independent risk factor for SDB. Disentangling the independent contribution of PCOS hormonal/metabolic features from obesity-related mechanisms remains challenging.

Narrative review methodology: This review did not employ systematic search protocols, formal quality assessment, or meta-analytic synthesis. Conclusions are therefore subject to selection bias and interpretive subjectivity. A summary table of key studies (Table 1) has been added to this revised version to improve transparency, as recommended by the reviewer.

## FUTURE RESEARCH DIRECTIONS

To address the substantial knowledge gaps identified in this review, the following research priorities are recommended:

1. Prospective cohort studies using full-night polysomnography with esophageal pressure (Pes) monitoring in well-phenotyped PCOS populations. Such studies should stratify by PCOS phenotype (hyperandrogenic, metabolic, ovulatory), BMI category, and age to identify subgroups at highest UARS risk. Standardized PCOS diagnostic criteria (Rotterdam or NIH) and AASM-defined RERA criteria should be employed.
2. Randomized controlled trials comparing CPAP vs. MADs vs. lifestyle intervention specifically for UARS in PCOS. Outcomes should include both sleep parameters (RDI, arousal index, sleep efficiency) and PCOS-specific metabolic and reproductive endpoints (insulin sensitivity, androgen levels, ovulation rates, quality of life).
3. Studies examining the impact of anti-androgen and insulin-sensitizing therapies on RERA frequency and sleep architecture. Do metformin, spironolactone, or oral contraceptives reduce UARS severity in PCOS? Mechanistic studies linking hormonal changes to upper airway physiology are needed.
4. Development and validation of PCOS-UARS-specific clinical prediction and screening tools. Broader clinical prediction models incorporating hormonal, metabolic, and anthropometric parameters (BMI, total testosterone, free androgen index) alongside symptom scores should be developed and prospectively validated. In parallel, existing questionnaire-based tools—including the STOP-BANG questionnaire, Berlin Questionnaire, Insomnia Severity Index, and Epworth Sleepiness Scale—should be validated in PCOS cohorts to identify high-risk patients for referral to sleep medicine without requiring Pes monitoring. This represents an immediately achievable near-term research priority.
5. Longitudinal studies assessing the impact of untreated UARS on long-term cardiometabolic outcomes in PCOS. Does UARS independently predict incident diabetes, hypertension, or cardiovascular events in PCOS cohorts? Such data would inform screening and treatment priorities.

## CONCLUSION

Upper Airway Resistance Syndrome represents a distinct and underrecognized SDB phenotype in women with polycystic ovary syndrome. The pathophysiological convergence of hyperandrogenism, progesterone deficiency, and insulin resistance is hypothesized to create heightened vulnerability to increased upper airway resistance and sleep fragmentation. Atypical clinical presentation in young non-obese women and diagnostic limitations of standard PSG contribute to systematic underdiagnosis.

Recognition of UARS in PCOS requires high clinical suspicion and, when available, esophageal pressure monitoring for accurate RERA detection. Before pursuing sleep evaluation, clinicians should systematically exclude common PCOS comorbidities that mimic UARS symptoms, including depression, hypothyroidism, and iron deficiency anemia (Table 2). Management necessitates integrated, multidisciplinary approaches addressing both metabolic-reproductive and respiratory dysfunction simultaneously. Early recognition and treatment of UARS in PCOS may mitigate long-term cardiometabolic risks and improve quality of life. Future prospective studies with esophageal pressure-based PSG are essential to establish true UARS prevalence and guide evidence-based management in this population. Increased awareness among gynecologists, endocrinologists, and otolaryngologists is essential to identify and appropriately manage this underrecognized condition.

## Data Availability

The datasets generated and/or analyzed during the current study are available from the corresponding author upon reasonable request.
